# The short-term and long-term outcomes of transcatheter or surgical aortic valve replacement in elderly patients: A protocol for a systematic review

**DOI:** 10.1097/MD.0000000000019307

**Published:** 2020-02-28

**Authors:** Xiang Lei, Zhili Wei, Shidong Liu, Fuxiang Liang, Bing Song

**Affiliations:** a First Clinical Medical College, Lanzhou University; b Department of Cardiovascular Surgery, First Hospital of Lanzhou University, Lanzhou, China.

**Keywords:** aortic stenosis, efficacy, safety, surgical aortic valve replacement, transcatheter aortic valve replacement

## Abstract

**Background:**

Transcatheter aortic valve replacement (TAVR) has become an essential alternate option for people suffering from aortic stenosis. However, the efficacy and safety of TAVR for elderly population (aged over 80 years) is still unclear.

**Methods:**

We plan to perform a systematic review and meta-analysis of clinical controlled trials and propensity-match cohort studies to evaluate the short- and long-term outcomes in elderly aortic stenosis patients who undergo a transcatheter or surgical aortic valve replacement. We will search PubMed, EMBASE, and Cochrane Library using a comprehensive strategy. The related conference proceedings and reference lists of the included studies will also be checked to identify additional studies. Two reviewers will screen retrieved records, extract information, and assess the risk of bias independently. STATA software will be used to conduct data synthesis. There is no requirement of ethical approval and informed consent.

**Results:**

This study will be submitted to a peer-reviewed journal for publication.

**Conclusion:**

This is the first systematic assessment of TAVR for elderly patients with aortic stenosis. We hope it will provide a relatively comprehensive reference for clinical practice and future relevant clinical trials.

**Ethics and dissemination:**

Ethics approval and patient consent are not required as this study is a systematic review and meta-analysis.

**PROSPERO registration number:**

CRD42019140857.

**Study protocol registry:**

The protocol has been registered in PROSPERO, which is an International Prospective Register of Systematic Reviews. The registration number is CRD42019140857

## 1 Introduction

Aortic stenosis (AS) is the most common native valve disease and primarily associated with advanced age, reportedly affecting up to nearly 10% of people aged over 80 years.^[1,2]^ Besides, according to the data from US Census Bureau, the elderly population is steadily increasing and is projected to double for octogenarian and become fourfold for nonagenarian over the next 2 or 3 decades. Obviously, an increasing incidence would be expected as life expectancy increases.^[3]^

Before the advent of transcatheter aortic valve replacement (TAVR), surgical aortic valve replacement (SAVR) was the only choice for aortic stenosis and has demonstrated improved survival and definitive treatment in symptomatic patients.^[4]^ However, a significant proportion of older enough people are not candidates for SAVR due to prohibitive surgical risk and associated comorbidities.^[5]^ Fortunately, last decade witnessed that TAVR has gradually become an effective alternative option for AS, with surgical indication changed from high-risk or inoperative patients to low- to intermediate-risk patients. Although current guidelines recommend that the choice for TAVR or SAVR should be dependent on various factors, particularly for patient-specific procedural risks, it is a common thing in clinical practice to refer patients aged >80 years to TAVR regardless of the individual's surgical risk because of their overall frailty and related comorbidities.

Currently, conflicting results have been reported by previous studies regarding the clinical outcomes between TAVR and SAVR in elderly people.^[6,7]^ To further confirm the efficiency and safety of TAVR in octogenarians and nonagenarians, we performed this systematic review and meta-analysis to explore the short- to long-term clinical outcomes of TAVR.

## 2 Methods

### 2.1 Protocol registration

The protocol has been registered in PROSPERO, which is an International Prospective Register of Systematic Reviews. The registration number is CRD42019140857 (http://www.crd.york.ac.uk/PROSPERO/).^[8]^ The content of this protocol will follow the preferred reporting items for systematic review and meta-analysis protocols (PRISMA-P) recommendations. We also plan to conduct it in accordance with the Cochrane Handbook for the Systematic Reviews of Interventions and Preferred Reporting Items for Systematic Review and Meta-Analysis (PRISMA) guidelines.^[9]^

### 2.2 Eligibility criteria

#### 2.2.1 Types of studies

Two-arm studies were conducted, including a randomized controlled trial (RCT) and a propensity-match cohort study (PSM) without published year, publication status limitations.

#### 2.2.2 Types of participants

Patients aged over 80 years with aortic stenosis were diagnosed by echocardiograph, CT, and MRI, requiring valve replacement. There were no restrictions on gender, STS risk score, EuroScore, type of valve, and access site.

#### 2.2.3 Types of interventions and comparators

The treatment group will be treated by TAVR, which is regarded as an effective treatment method for severe AS patients at prohibitive, high, and intermediate surgical risk. The control group will be treated with SAVR, which is the predominant treatment method for severe AS patients.

#### 2.2.4 Types of outcome measures

Outcomes were mainly identified by relevant literature and existing clinical practice. The primary outcome is all-cause mortality in 30 days, 1 year, 2 years, and 5 years. The secondary outcomes include transient ischemic attack (TIA), stroke, rehospitalization, myocardial infarction, major vascular complication, life-threatening or disabling bleeding, acute kidney injury, new atrial fibrillation, new permanent pacemaker, moderate/severe paravalvular regurgitation, NYHA class III or IV, aortic valve orifice area, aortic valve mean gradient, and left ventricle ejection fraction (LVEF). Besides, all the endpoints reported in the included studies will be collected and evaluated, although we may not mention some of them in this protocol.

### 2.3 Literature search

A systematic search of the literature will be conducted without language and year restrictions to identify all relevant clinical controlled trials or propensity-match cohort studies. We will search following electronic databases: PubMed, EMBASE, and Cochrane Library from 2002 to October 2019 using related search terms, including “transcatheter aortic valve replacement’,” “nonagenarian,” and “octogenarian.” In addition, Congress and conference proceedings will be manually retrieved. Related articles and references of included research will also be tracked to find potential studies. If significant data was incomplete in included study, we will contact the authors to get unpublished data.

### 2.4 Study selection and data extraction

After being imported into Endnote X7 and duplication, retrieved records will be independently screened by two reviewers (LX and WZL). First, we will read the titles and abstracts of all indentified records to exclude clearly unrelated records based on the inclusion criteria. Then the full texts of the articles retained will be reviewed to further determine their suitability. Any disagreement will be resolved by a third reviewer (LFX or SB). We will show the selection process in detail in the PRISMA flow chart.^[10]^

Two authors (LX and WZL) of this review will independently extract the data using a pre-defined form. The basic characteristics, related outcome, and quality evaluation information of included studies will be collected. Similarly, any discrepancies will be resolved by a third reviewer (LFX or SB). Data extracted will include author, year, study type, number of participants, intervention, control, demographics, complications, previous history, cardiac ultrasound data, NYHA class, STS risk score, EuroScore, type of valve, and follow-up time. The incidence of any cause of death, TIA, stroke, rehospitalization, myocardial infarction, major vascular complication, life-threatening or disabling bleeding, acute kidney injury, new atrial fibrillation,new permanent pacemaker and moderate/severe paravalvular regurgitation, the change of NYHA class III or IV, and the data of aortic-valve area, mean gradient and LVEF.

### 2.5 Quality of evidence assessment

The quality of included studies will be assessed by Grading of Recommendations Assessment Development and Evaluation (GRADE), and divided into 4 levels: high quality, moderate quality, low quality, and very low quality.^[11]^

### 2.6 Assessment of study bias

Included study bias will be independently assessed by two reviewers (LX and LSD) and any disagreement will be solved by a third reviewer (LFX or SB). For randomized controlled trials, we will use the Cochrane risk of bias tools to evaluate potential bias in seven specific domains:

1. sequence generation,
2. allocation concealment,
3. blinding of participants and personnel,
4. blinding of outcome assessment,
5. incomplete outcome data,
6. selective outcome reporting,
7. other bias.^[12]^

For propensity-match cohort studies, 9-star Newcastle-Ottawa Scale will be applied, which rates studies based on 8 criteria in 3 sources of bias.^[13]^

### 2.7 Statistical analysis

For dichotomous variables, the relative risk (RR) with 95% confidence intervals (CI) were calculated from each study. Continuous variables will be presented as standard mean difference (SMD) with 95% CI. All endpoints will be combined and meta-analysis will be performed by using DerSimonian and Laird random effects model.^[14]^ We assessed statistical heterogeneity by using Chi-Squared test and I^2^ statistic. We will consider significant heterogeneity when *P* < .10 for Chi-Squared or I^2 ^> 50%.^[15]^ All primary analyses were performed with STATA v15.1 (Stata Corp, College Station, TX).

#### 2.7.1 Subgroup analysis

We will also conduct subgroup analysis to find more potential information based on pre-set criteria in four variables:

1. different study design, RCT, or PSM;
2. different follow-up time;
3. patients in different STS surgical risk score;
4. different types of implanted valve in TAVI patients.

#### 2.7.2 Sensitivity analysis

If the heterogeneity is high, we will conduct sensitivity analyses based on the study type, follow-up time, and types of valve.

#### 2.7.3 Publication bias

The likelihood of publication bias was assessed graphically through the generation of funnel plots, evaluated using an Egger test.^[16]^

## 3 Results

The study does not require ethical approval because the meta-analysis is based on published research and the original data are anonymous. And this study will eventually be published in a peer-reviewed journal in the form of a scientific paper.

## 4 Discussion

To our knowledge, this is the first systematic review and meta-analysis concerning the short- and long-term outcomes of transcatheter or surgical aortic valve replacement in patients aged over 80 years. The results from our research may provide meaningful evidence for clinical practice and give a valuable reference for future study.

There seem to be some potential limitations for our study. Firstly, we only include English language articles, which might miss some important data in other-language articles. In addition, according to the initial search result, less random controlled trials and more propensity-match cohort studies will be included in our study, which may have an obstacle to our data pooling and results interpretation. But it will probably help to promote several more reliable conclusions and focus on more precious direction for future clinical studies to some extent. We hope to provide a prompt and credible evaluation for elderly people who plan to undergo a TAVR or SAVR.

## Author contributions

**Data curation:** Xiang Lei and Zhili Wei

**Methodology:** Fuxiang Liang and Shidong Liu

**Project administration:** Bing Song

**Writing – original draft:** Xiang Lei and Zhili Wei

**Writing – review &editing:** Fuxiang Liang and Bing Song
